# The Methyl-CpG-Binding Protein Mbd2 Regulates Susceptibility to Experimental Colitis via Control of CD11c^+^ Cells and Colonic Epithelium

**DOI:** 10.3389/fimmu.2020.00183

**Published:** 2020-02-14

**Authors:** Gareth-Rhys Jones, Sheila L. Brown, Alexander T. Phythian-Adams, Alasdair C. Ivens, Peter C. Cook, Andrew S. MacDonald

**Affiliations:** ^1^Faculty of Biology, Medicine and Health, Manchester Collaborative Centre for Inflammation Research, Lydia Becker Institute of Immunology and Inflammation, University of Manchester, Manchester, United Kingdom; ^2^Manchester Academic Health Science Centre, Manchester, United Kingdom; ^3^Centre for Inflammation Research, The Queen's Medical Research Institute, University of Edinburgh, Edinburgh, United Kingdom; ^4^Centre for Immunity, Infection and Evolution, School of Biological Sciences, Institute of Immunology and Infection Research, University of Edinburgh, Edinburgh, United Kingdom

**Keywords:** epigenetics, colitis, macrophage, epithelium, dendritic cell

## Abstract

Methyl-CpG-binding domain-2 (Mbd2) acts as an epigenetic regulator of gene expression, by linking DNA methylation to repressive chromatin structure. Although Mbd2 is widely expressed in gastrointestinal immune cells and is implicated in regulating intestinal cancer, anti-helminth responses and colonic inflammation, the Mbd2-expressing cell types that control these responses are incompletely defined. Indeed, epigenetic control of gene expression in cells that regulate intestinal immunity is generally poorly understood, even though such mechanisms may explain the inability of standard genetic approaches to pinpoint the causes of conditions like inflammatory bowel disease. In this study we demonstrate a vital role for Mbd2 in regulating murine colonic inflammation. *Mbd2*^−/−^ mice displayed dramatically worse pathology than wild type controls during dextran sulfate sodium (DSS) induced colitis, with increased inflammatory (IL-1β^+^) monocytes. Profiling of mRNA from innate immune and epithelial cell (EC) populations suggested that Mbd2 suppresses inflammation and pathology via control of innate-epithelial cell crosstalk and T cell recruitment. Consequently, restriction of Mbd2 deficiency to CD11c^+^ dendritic cells and macrophages, or to ECs, resulted in increased DSS colitis severity. Our identification of this dual role for *Mbd2* in regulating the inflammatory capacity of both CD11c^+^ cells and ECs highlights how epigenetic control mechanisms may limit intestinal inflammatory responses.

**Graphical Abstract:**
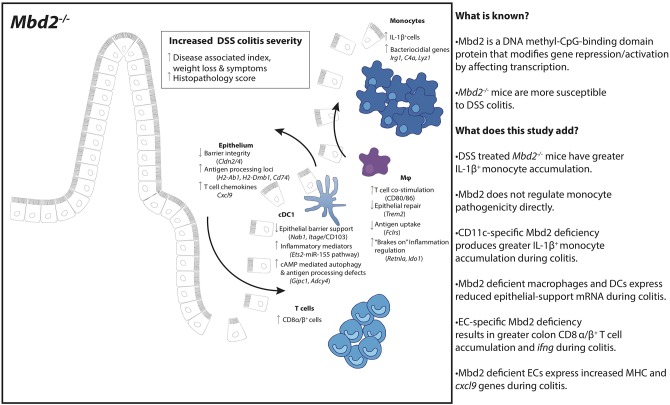
Overview of Mbd2 deficient mice during DSS colitis.

## Introduction

Epigenetic mediated changes in gene expression that are not encoded in DNA sequence, and thus not accounted for using existing GWAS, represent an attractive mechanism for explaining part of inflammatory bowel disease (IBD) susceptibility (1). Epigenetic processes such as DNA methylation, histone modification and nucleosome remodeling have all been shown to influence the regulation of key cell functions, including the immune response, and require the presence of methyl-binding domain proteins (Mbds) to exert these effects efficiently (2, 3). One of these, Mbd2, binds to methylated DNA, recruiting a nucleosome remodeling deacetylase (NuRD) complex that can significantly influence gene expression (3). Mbd2 has been shown to play an important role in gastrointestinal (GI) tract response to helminths and in predisposition to colorectal malignancy (4, 5). In addition, we have shown that Mbd2 deficient intestinal T cells over-express IFN-γ in experimental colitis (6), that Mbd2 deficient mice develop chronic intestinal inflammation after a single mucosal injury (6), and Mbd2 deficient dendritic cells (DCs) are less able to initiate immune responses (2). However, the mechanisms underpinning these observations, and the range of GI tract mucosal cells under the control of Mbd2, are poorly described.

Innate immune cells, particularly macrophages and DCs, are essential to maintain a balanced response in the intestines during health and inflammation. Macrophages, abundant in the gut mucosa (identified as CD11b^+^F4/80^+^CD64^+^MHC-II^+^Ly6C^−^{mice} or CD14^+^CD64^+^HLA-DR^Hi^SIRPα^+^{human}), are poorly responsive to Toll-like receptor (TLR) stimulation and secrete large quantities of the regulatory cytokine IL-10 in the steady state (7). However, this phenotype is disrupted during inflammation, where macrophages are capable of pro-inflammatory cytokine release that is in part controlled by histone reader facilitated regulation of NF-kB response elements (8).

Like macrophages, DCs are central to intestinal homeostasis, with several intestinal subsets which differ in origin, development and function (9). Murine cDC1s (identified as CD11b^−^CD103^+^XCR1^+^), equivalent to CD103^+^Sirpα^−^ DCs in humans, are dependent on transcription factors (TFs) Id2, Irf8, and Batf3 and induce anti-viral/bacterial IFNγ (Th1) CD4^+^ T cell responses (10). In contrast, CD11b^+^CD103^+^ intestinal cDC2s are related to CD103^+^Sirpα^+^ DCs in human intestines, depend on TFs Irf4 and Notch2 (10) and are important for induction of IL-17 (Th17) and IL-4/IL-5/IL-13 (Th2) CD4^+^ T cell responses (11). Whether epigenetic gene regulation is important in controlling immune responses of these subsets in the gut is unknown. However, inhibition of histone reading proteins in DCs *in vitro* causes failure of DC maturation and DC-mediated antigen dependent proliferation of naïve T cells (12, 13). Therefore, epigenetic regulation of gene expression within these DC subsets is likely to be crucial for their functional capability of mediating intestinal immunity.

In addition to DCs and macrophages, colonic epithelial cells (CECs) play a key role in barrier integrity and immune responses. ECs develop from pluripotent stem cells in the crypt niche, functional plasticity of which is dependent upon epigenetic proteins such as polycomb protein-mediated changes in histone modification. Indeed, altered histone motifs via HDAC1 and 2 inhibition cause barrier failure and susceptibility to colitis (14). ECs also express anti-microbial products (such as calprotectin and defensins), and may facilitate presentation of antigen via MHC-I and -II (15), so are poised to co-ordinate downstream immune responses, which may be in part reliant on epigenetic control (4, 16).

In this work, we have discovered that Mbd2 acts as a central regulator of intestinal inflammation. We found that the severe inflammation that develops in *Mbd2*^−/−^ mice during DSS driven colitis (6) is accompanied by a large accumulation of IL-1β^+^ monocytes. Although mRNA analysis of isolated cells from *Mbd2*^−/−^ mice revealed no intrinsic role for Mbd2 in colon monocyte gene regulation, this approach identified dysregulated cDC1 and macrophage expression of genes linked to innate cell-CEC crosstalk and intestinal homeostasis. Strikingly, restriction of Mbd2 deficiency to CD11c^+^ cells resulted in an exacerbated IL-β^+^ monocyte mediated colitis. Further, *Mbd2*^−/−^ CECs displayed profound dysregulation of genes controlling MHC, with EC-restricted Mbd2 deficiency resulting in elevated intestinal CD8^+^ T cell responses. These data reveal an epigenetic mechanism that is central to limiting excessive colonic inflammation via two discrete processes and identify methyl-CpG-binding proteins and the genes under their control as potential therapeutic targets for intestinal inflammatory disease.

## Results

### *Mbd2* Is a Central Regulator of Susceptibility to Colonic Inflammation

**Figure 1:**
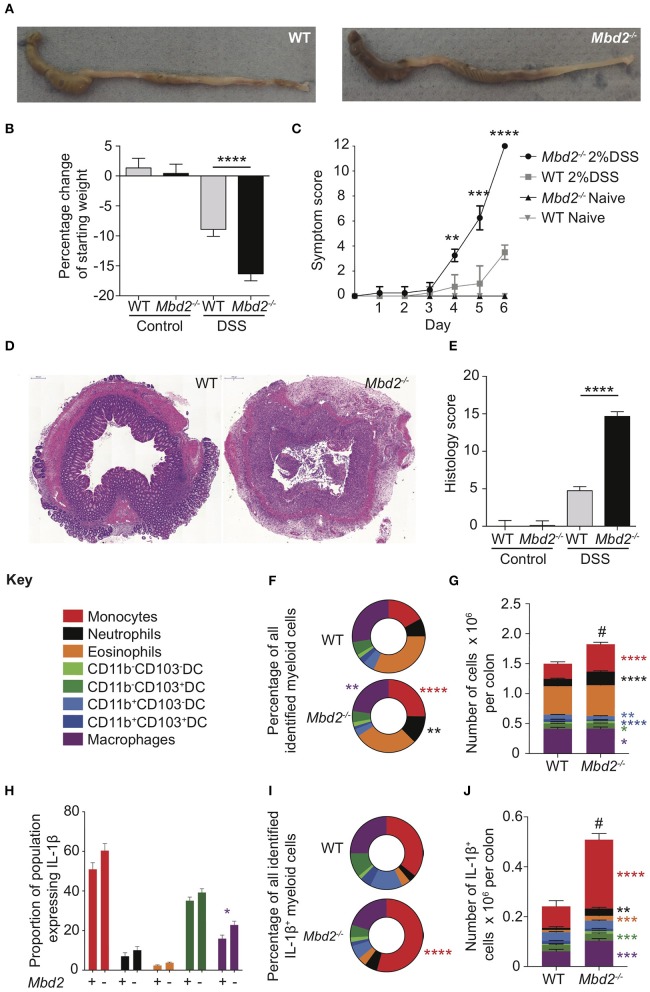
Expression of *Mbd2* is vital to limit the severity of pathology during colitis. *Mbd2*^−/−^ or littermate WT mice were co-housed and subjected to DSS in drinking water, tissues were harvested 6 days post consecutive treatment. The severity of colitis in the mice was assessed by **(A)** representative colon gross pathology, **(B)** weight loss following DSS treatment, and **(C)** symptom score (calculated from daily assessment of weight loss, rectal bleeding and stool consistency). **(D,E)** colon sections obtained from the mice were stained with H&E and colitis severity was assessed blinded according to a DSS histo-pathology score. **(F,G)** Colonic lamina propria cells were isolated and the relative proportion and number of monocytes, neutrophils, eosinophils, macrophages, and DC subsets were assessed by flow cytometry. Lamina propria cells were incubated for 3 h with 1 μl/ml Golgistop and **(H)** the proportion of monocyte, neutrophil, eosinophil, cDC1s (CD11b^−^CD103^+^) and macrophages that express IL-1β **(I)** the proportion of myeloid IL-1β^+^ cells **(J)**, and the total number of IL-1β^+^ cells was assessed by intracellular staining and flow cytometry. Mean symptom score ± SEM, representative data of 3 independent experiments **(C)** presented, all other graphs show least mean square + SEM, *n* = 15–25 per group, analyzed by linear regression of 6 independent experiments. *P < 0.05, **P < 0.01, ***P < 0.001, ****P < 0.0001, # comparison of total number of myeloid cells DSS treated *Mbd2*^−/−^ vs. WT (*P* < 0.0001).

Assessment of Mbd2 distribution throughout the murine small and large intestine using RT-qPCR showed that *Mbd2* mRNA expression was higher in the large vs. small intestine, and greater in the distal (rectum) vs. proximal (caecum) colon (Supplementary Figure 1a). In addition, *MBD2* mRNA levels were significantly reduced in active human IBD (Supplementary Figure 1b). This tightly controlled GI tract *Mbd2* expression suggested that it may be an important regulator of colon inflammation. To address this possibility, we investigated how *Mbd2* deficiency affected the colonic response to inflammation. Naïve *Mbd2*^−/−^ mice do not develop spontaneous intestinal inflammation (17), maintained an intact epithelial barrier and had equivalent levels of colon *ifng*/IFN-γ to WT controls (Supplementary Figures 1c–e). Murine ingestion of DSS causes intestinal barrier breakdown, exposing the underlying mucosa to the microbiota, with resultant tissue damage, diarrhea, weight loss, and rectal bleeding (17). In keeping with our previous observations (6), the colons of DSS treated *Mbd2*^−/−^ mice displayed significantly worse pathology than WT controls, with increased colon shortening, weight loss and histopathology (Figures 1A–E).

As the role of Mbd2 in myeloid cells in intestinal inflammation is not known, we used multi-parameter flow cytometry (gating strategy defined in Supplementary Figure 2) to assess these populations in the colon lamina propria (LP). Proportions of myeloid cell populations from naïve *Mbd2*^−/−^ colon LP were similar to WT mice (Supplementary Figures 3a,b). Upon DSS challenge, the LP myeloid compartment of *Mbd2*^−/−^ mice had a significantly greater proportion of monocytes (Ly6C^+^CD11b^+^MHC-II^±^) and neutrophils (CD11b^+^Ly6G^+^), and less macrophages, compared to WT (Figure 1F). In addition, total numbers overall of monocytes, neutrophils, macrophages, and cDC1s were increased in *Mbd2*^−/−^ vs. WT (Figure 1G).

Myeloid cell secretion of pro-inflammatory cytokines, particularly IL-1β, is a key factor in mediating intestinal inflammation, with monocytes often a major source of this cytokine (18). Analysis of colon myeloid cell *ex vivo* cytokine production showed that naïve *Mbd2*^−/−^ mice had equivalent IL-1β producing capabilities to WT as assessed by per cell, total cell number, and overall proportion (Supplementary Figures 3c–e). After DSS treatment, only *Mbd2*^−/−^ macrophages demonstrated increased per cell IL-1β production (Figure 1H). However, as *Mbd2*^−/−^ LP displayed a marked proportional increase in monocytes in response to DSS (Figures 1F,G), these cells became the dominant source of IL-1β^+^ (Figure 1I). Moreover, as myeloid cell numbers were greater in *Mbd2*^−/−^ mice, increased total numbers of IL-1β^+^ monocytes, neutrophils, eosinophils, cDC1s, and macrophages were evident in the LP of DSS-treated *Mbd2*^−/−^ colon (Figure 1J). Thus, *Mbd2* was required to prevent increased colonic inflammation involving augmented weight loss, diarrhea, pan colitis, tissue architecture destruction, and an immune cell infiltrate characterized by pro-inflammatory cytokine secreting monocytes and neutrophils.

### *Mbd2* Deficiency in Monocytes Is Not Associated With a Pro-inflammatory Transcriptome

**Figure 2:**
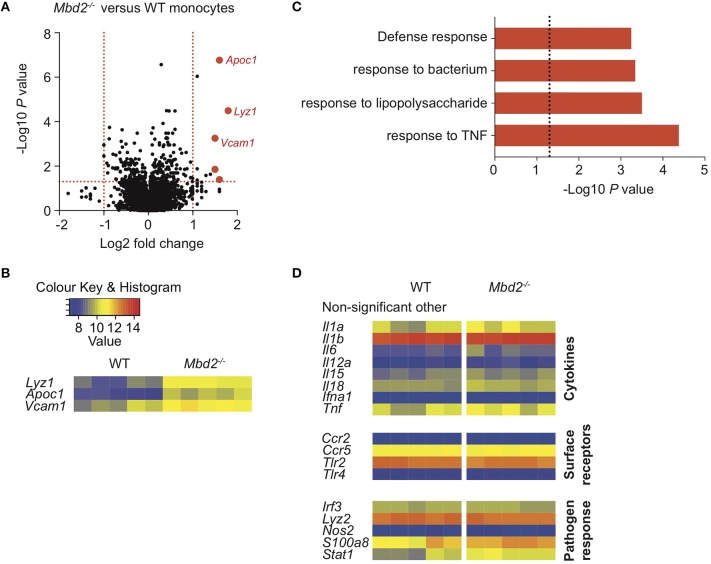
The impact of Mbd2 deficiency on mRNA expression by colon monocytes during colitis. Monocytes (CD11b^+^Ly6C^+^F4/80^−^MHC-II^−/+^) were isolated and purified via FACS from the colon lamina propria of *Mbd2*^−/−^ or WT littermate co-housed mice after 6 consecutive days of DSS treatment. RNA was extracted for microarray mRNA expression analysis (5 biological replicates per genotype). **(A)** Volcano plot depicts differential gene expression profile of *Mbd2*^−/−^ vs. WT monocytes, with selected genes highlighted in red. Dashed lines represent *P* < 0.05, and ± 1-fold change. **(B)** Heat map of relative expression values for the highlighted loci in **(A)** (log_2_ normalized intensity, one-fold change-filtered, *P* < 0.05). **(C)** Selected pathways from GOterm analysis of significantly altered mRNA transcripts (*P* < 0.05) from **(A)**, dashed line represents *P* < 0.05. **(D)** selected other non-significant loci based on literature review of monocyte-associated inflammatory processes.

In mice, LP monocytes have similar marker expression to blood monocytes (CD33, CD64, CD16, CX3CR1) but are potent producers of pro-inflammatory cytokines IL-1β, IL-6, MMP-1 and MMP-9 after stimulation with LPS, compared to other monocyte subsets (19). Given the importance of these cells in promoting inflammatory responses, and our observed increase in IL-1β^+^ monocytes in *Mbd2*^−/−^ mice (Figure 1), we investigated whether Mbd2 plays an intrinsic role in regulating monocytes by analyzing the mRNA expression of purified colon monocytes from DSS treated *Mbd2*^−/−^ and WT mice. In total, only 7 genes (including *Apoc1, Lyz1*, and *Vcam1*, which encode apolipoprotein C1, the bacteriocidal enzyme lysozyme and the adhesion molecule Vcam1) were significantly dysregulated (Log_2_ fold change ± 1, *P* < 0.05, all upregulated) when comparing *Mbd2*^−/−^ monocytes to WT controls (Figures 2A,B). Considering all significant (*P* < 0.05) genes irrespective of fold change, GO term enrichment revealed upregulated pathways in *Mbd2*^−/−^ vs. WT monocytes that included “response to TNF,” “response to LPS,” “response to bacterium,” and “defense response” in the top10 most enriched items (Figure 2C). However, the vast majority of loci associated with monocyte pro-inflammatory ability (e.g., *Il1a, Il1b, Il6*, and *Tlr2*) were unaffected by *Mbd2* deficiency (Figure 2D). This suggests that the elevated monocyte numbers in *Mbd2*^−/−^ may not be due to Mbd2 intrinsic regulation of their function and may instead be a consequence of Mbd2 deficiency altering their recruitment or retention in response to DSS.

### *Mbd2* Deficiency in CD11c^+^ Cells Confers Increased Susceptibility to Colonic Inflammation

We reasoned that influx of neutrophils and monocytes to the colon might be limited by upstream cell populations under the influence of Mbd2 mediated gene regulation. Macrophages and DCs have both been implicated in DSS pathogenesis (20, 21) with macrophages an important source of monocyte chemokines such as CCL8 (22). Of the DC subsets, CD11b^−^CD103^+^ cDC1s which express *bona fide* DC TFs (23) and are crucial for DSS pathogenesis via CEC crosstalk (24), were the only expanded DC population in the absence of Mbd2 (Figure 1G). Similarly, macrophages were the most numerous LP myeloid population (Supplementary Figure 3b) and the only myeloid cell to significantly increase per cell production of IL-1β after DSS in Mbd2 deficiency (Figure 1H).

**Figure 3:**
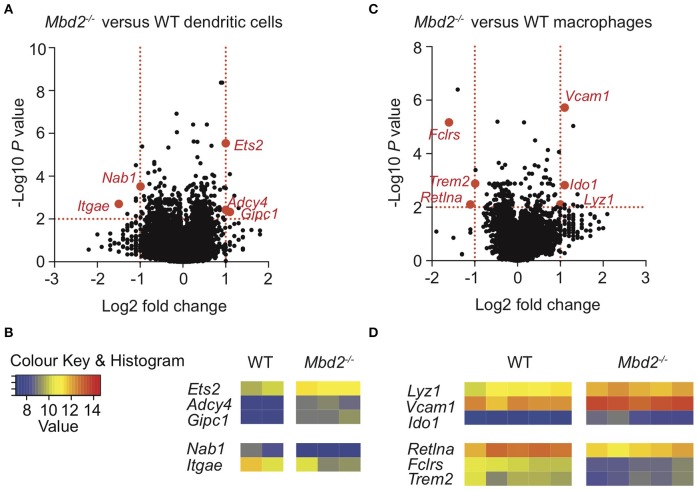
Mbd2 regulates expression of both pro- and anti-inflammatory genes in cDC1 and macrophage populations during colitis. Colon lamina propria cDC1s (CD11c^+^CD11b^−^CD103^+^) **(A,B)** and macrophages (CD11b^+^F4/80^+^MHC-II^+^Ly6C^−^) **(C,D)** were isolated from *Mbd2*^−/−^ or WT littermate co-housed mice after 6 consecutive days of DSS treatment with RNA extracted for microarray mRNA expression analysis (2–5 biological replicates per genotype). **(A,C)** Volcano plot depicts differential gene expression profile of *Mbd2*^−/−^ vs. WT cDC1s **(A)** or macrophages **(C)**, with selected genes highlighted in red. Dashed lines represent *P* < 0.05, and ±1-fold change. **(B,D)** Heat map of relative gene expression values from those highlighted loci in **(A,C)** (log_2_ normalized intensity, one-fold change-filtered, *P* < 0.05).

Therefore, to understand whether cDC1 or macrophage Mbd2 deficiency was important for the increased susceptibility of *Mbd2*^−/−^ mice to DSS colitis, we performed microarray analysis on purified CD11b^−^CD103^+^ DCs and macrophages from WT and *Mbd2*^−/−^ DSS treated mice. cDC1s from DSS treated *Mbd2*^−/−^ mice showed 27 significantly dysregulated genes (Log_2_ fold change ± 1, *P* < 0.05, 10 up-, and 17 down-regulated) vs. WT controls (Figures 3A,B). *Ets2*, the most significantly upregulated locus in *Mbd2*^−/−^ cDC1s, is a transcriptional regulator that increases expression of miR-155, a potent pro-inflammatory mediator found at increased levels in IBD mucosa (25, 26). *Gipc1* and *Adcy4*, also significantly upregulated in *Mbd2*^−/−^ DCs, encode a GTPase activator protein for Gαi/Gαq and adenylate cyclase enzyme, respectively, both of which can increase DC cAMP levels (27). Of the genes downregulated in *Mbd2*^−/−^ DCs, *Nab1*, and *Itgae* (CD103) may affect CEC function by reducing STAT5 and E-cadherin signaling, respectively, that may pre-dispose to epithelial barrier breakdown (28, 29). Considering all significant (*P* < 0.05) genes irrespective of fold change, GOterm pathway analysis revealed no immunological pathways of note, though *Ets2, Gipc1*, and *Adcy4* were all represented in the “intracellular” pathway, which was also the most enriched GOterm (*P* = 5.1 × 10^−7^) (Supplementary Table 1).

Isolation and mRNA microarray analysis of macrophages showed 41 significant genes were dysregulated (Log_2_ fold change ± 1, *P* < 0.05, 38 up-, and 3 down-regulated) in DSS treated *Mbd2*^−/−^ mice, compared to WT (Figures 3C,D). *Mbd2*^−/−^ colon macrophages displayed altered expression of genes associated with both dampening pro-inflammatory responses (*Retnla* and *Ido1*) and promoting bactericidal pathways (*Lyz1*), in addition to epithelial wound healing (*Trem2*), vascular adhesion (*Vcam1*, as also seen in Mbd2 deficient monocytes), and *Fclrs* (a poorly described gene that has putative scavenger binding domains) (30–33). However, as in DC GO term analysis, there were no significantly dysregulated pathways of interest (Supplementary Table 2).

**Figure 4:**
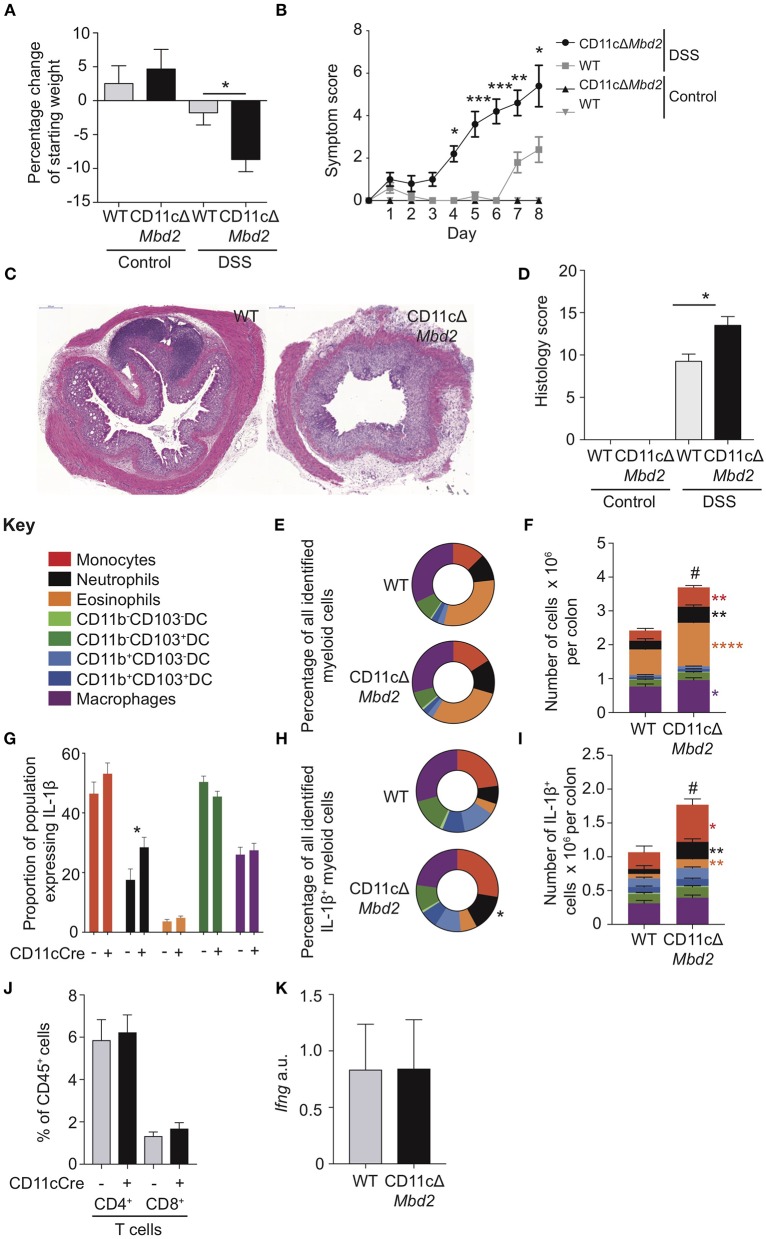
CD11c^+^ cell expression of Mbd2 is essential to limit colitis. CD11cΔ*Mbd2* or littermate *Cre*^−^ (WT) mice were co-housed and tissues were harvested 8 days post consecutive treatment DSS treatment. The severity of colitis in the mice was assessed by **(A)** weight loss following DSS treatment and **(B)** symptom score (calculated from daily assessment of weight loss, rectal bleeding, and stool consistency). **(C)** colon sections obtained from DSS treated mice were stained with H&E and **(D)**, colitis severity was assessed blinded according to a DSS histo-pathology score. Colonic lamina propria cells were isolated and the relative proportion and number of monocytes, neutrophils, eosinophils, macrophages DC subsets **(E,F)** and T cells **(J)** were assessed by flow cytometry. Lamina propria cells were incubated for 3 h with 1 μl/ml Golgistop and **(G)** the proportion of monocyte, neutrophil, eosinophil, cDC1s and macrophages that express IL-1β **(H)** the proportion of myeloid IL-1β^+^ cells, **(I)** and the total number of IL-1β^+^ cells was assessed by intracellular staining and flow cytometry. **(J)** Number of CD4^+^ and CD8α/β^+^ TCRα/β T cells as a proportion of CD45^+^ cells. **(K)** *ifng* mRNA levels derived from 1 cm sections of distal colon in DSS treated CD11cΔ*Mbd2* or littermate *Cre*^−^ mice determined by qPCR relative to *gapdh*. Mean symptom score ± SEM, representative data of 3 independent experiments **(B)** presented, all other graphs show least mean square + SEM, *n* = 15–25 per group, analyzed by linear regression of 6 independent experiments, except **(J,K)** which are representative data from 3 independent experiments. *P < 0.05, **P < 0.01, ***P < 0.001, ****P < 0.0001. # comparison of total number of myeloid cells DSS treated CD11cΔ*Mbd*2 vs. *Cre*^−^ controls (*P* < 0.0001).

Both macrophages and DCs in the colon express the integrin CD11c, and depletion of CD11c^+^ cells using CD11c-DTR mice may reduce or increase colitis severity, depending on the presence or absence of the TLR9 agonist CpG (20, 21). We thus restricted *Mbd2* deficiency to CD11c expressing cells using a *CD11c-Cre*^+^ *Mbd2*^*Fl*/*Fl*^ system (CD11cΔ*Mbd2*) (2). Although CD11c was expressed by a range of cell types in the colon (Supplementary Figures 4a,b), >75% of colon CD11c^+^ cells were either DCs (25%) or macrophages (50%), with DC's displaying the greatest per cell expression level (Supplementary Figures 4a,b). Naïve CD11cΔ*Mbd2* colon LP displayed equivalent myeloid cell proportions, total cell number and per cell IL-1β production, total IL-1β^+^ cell number and overall proportion of IL-1β^+^ myeloid populations to *Cre*^−^ control mice (Supplementary Figures 5a–e). However, CD11cΔ*Mbd2* mice were significantly more susceptible to DSS colitis than *Cre*^−^ controls, displaying greater weight loss, symptom score, and tissue architecture destruction (Figures 4A–D). Due to an increased cellular infiltrate in CD11cΔ*Mbd2* DSS treated animals the number of monocytes, neutrophils, eosinophils and macrophages was greater compared to DSS treated *Cre*^−^ mice (Figures 4E,F). Measurement of *ex vivo* IL-1β production by myeloid cells showed that this was equivalent for all populations in CD11cΔ*Mbd2* and *Cre*^−^ DSS treated mice, with the exception of neutrophils (Figure 4G). As such, the myeloid compartment was enriched for IL-1β^+^ neutrophils in CD11cΔ*Mbd2* vs. *Cre*^−^ DSS treated mice (Figure 4H). Overall there were significantly more IL-1β^+^ cells in CD11cΔ*Mbd2* vs. *Cre*^−^ controls after DSS, increased in neutrophils, monocytes and eosinophils (Figure 4I). As *Itgae* (CD103) downregulation was one the most significant effects of Mbd2 deficiency in cDC1s (Figures 3A,B), we assessed surface levels of CD103 on cDC1s (CD11b^−^CD103^+^) and cDC2s (CD11b^+^CD103^+^) isolated from DSS treated *Mbd2*^−/−^, CD11cΔ*Mbd2*, or control mice (Supplementary Figure 6). In support of our microarray data (Figure 3), CD103 expression showed a small but significant reduction on colon LP cDC1s and cDC2s from *Mbd2*^−/−^ mice (Supplementary Figures 6a,b). In addition, when Mbd2 deficiency was restricted to CD11c^+^ cells, a subtle reduction in cDC1 CD103 expression was still observed (Supplementary Figures 6c,d). Lastly, in contrast to our previous work revealing a role for IFN-γ producing CD4^+^ and CD8^+^ T cells in mediating colitis susceptibility in global Mbd2 deficient mice (6), the total number of colon LP CD4^+^ or CD8^+^ T cells and colon IFN-γ mRNA expression were equivalent between DSS treated CD11cΔ*Mbd2* mice and controls (Figures 4J,K), suggesting increased colitis susceptibility was T cell independent in this context.

Collectively, these data show that CD11c-restricted Mbd2 deficiency resulted in increased severity of experimental colitis, characterized by elevated infiltration of IL-1β producing neutrophils and monocytes but equivalent numbers of LP T cells and colon IFN-γ transcript. Colon cDC1s and macrophages isolated from DSS treated *Mbd2*^−/−^ mice also displayed altered expression of genes associated with epithelial crosstalk (*Trem2, Nab1, Itgae*) and inflammation control (*Retlna, Ido1, Lyz1*). However, the increased inflammation evident in DSS exposed CD11cΔ*Mbd2* mice was less dramatic that in similarly treated global *Mbd2*^−/−^ mice (Figures 1A–J). This suggested that, while *Mbd2* expression in CD11c^+^ cells was important to limit pathology, it also regulated gene expression in other, CD11c negative, cell types to dampen inflammation during colitis.

### *Mbd2* Deficiency in Colon Epithelial Cells Confers Increased Susceptibility to Colonic Inflammation

Given that restriction of *Mbd2* deficiency to CD11c^+^ cells did not result in as severe inflammation as that seen in global *Mbd2*^−/−^ animals, and DSS is characterized by intestinal epithelial damage (34), we speculated that CECs may represent an important cell type that could be controlled by *Mbd2* during colitis.

**Figure 5:**
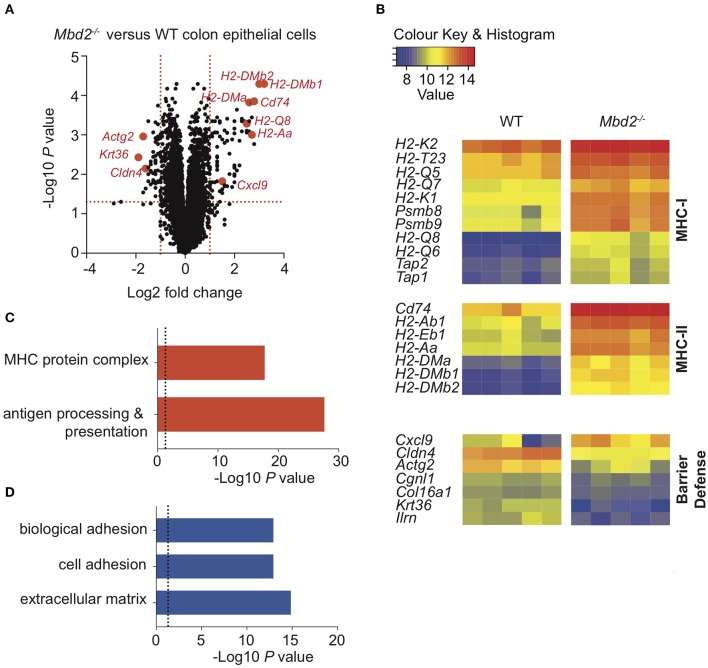
The impact of Mbd2 deficiency on mRNA expression by colon epithelial cells during colitis. **(A)** Volcano plot depicts differential gene expression profile of mRNA as determined by microarray of colon epithelial cells (CD45^−^ Lin^−^ F4/80^−^ CD3^−^ EpCAM^+^) isolated by flow cytometry after 6 consecutive days DSS treatment in *Mbd2*^−/−^ or WT littermate co-housed mice. **(B)** Heat map of relative gene expression values from selected significant (log_2_ fold change ± 1, *P* < 0.05) loci and selected up **(C)** and down **(D)** (*P* < 0.05) pathways from GOterm analysis from **(A)** (log_2_ normalized intensity, one-fold change-filtered, *P* < 0.05), 5 biological replicates per genotype.

To test this, we identified an EpCAM^+^CD45^−^Lin^−^ population of CECs [Supplementary Figure 7a, (35)] and analyzed mRNA expression of FACS-purified cells by qPCR. This revealed expression of EC-specific genes [intestinal stem cell niche (*Lrg5*), goblet cell (*Muc2*), enteroendocrine (*ChgA)*, and colonocyte (*Car1*)] in addition to high levels of *Mbd2* (Supplementary Figure 7b). To determine whether Mbd2 was critical for CEC regulation during inflammation, CECs were isolated from DSS treated WT and *Mbd2*^−/−^ mice and mRNA expression assessed by microarray. Comparison of *Mbd2*^−/−^ to WT CECs revealed that 118 genes were significantly dysregulated (Log_2_ fold change ± 1, *P* < 0.01, 75 up-, and 43 down-regulated), a more dramatic effect than was evident in myeloid cells (Figure 5A and Supplementary Figure 8). In CECs from DSS treated *Mbd2*^−/−^ mice, there was a striking up-regulation of MHC-I (including *H2-Q8, H2-K2*, and *H2-Q6*), MHC-II (including *H2-DMb1, H2-DMb2*, and *H2-DMa*), other MHC-related genes (including *Cd74, Ciita, Tap1/2*, and *Psmb8/9*), and the T cell chemoattractant *Cxcl9* (Figures 5A,B). At the same time, a significant decrease in levels of expression of genes that promote epithelial barrier integrity was also apparent (*Cldn4, Krt36*, and *Actg2*) (Figures 5A,B). Considering all significant (*P* < 0.05) genes irrespective of fold change, GO term enrichment revealed 366 significantly up-regulated and 425 significantly down-regulated array features, comparing *Mbd2*^−/−^ vs. WT CECs, with numerous immune cell function related pathways including “antigen processing & presentation,” and “MHC protein complex,” (up-regulated) and “cell adhesion,” “biological adhesion,” and “extracellular matrix” (down-regulated) in the top10 most enriched terms (Figures 5C,D). Together, this reveals that *Mbd2* plays a dominant role in controlling CEC mRNA expression during intestinal inflammation.

We have previously shown that Mbd2 deficiency in ECs using an inducible *Cre* model does not increase susceptibility to colitis (6). However, we hypothesized this was due to a modest reduction in EC Mbd2 expression using this approach (~20% reduction compared to WT levels), and that further reductions in Mbd2 expression may be biologically significant, given the profoundly altered transcriptome we had observed in Mbd2-deficient CECs (Figure 5). Therefore, we investigated CEC-restricted *Mbd2* deficiency using a constitutive *Villin-Cre*^+^ *Mbd2*^*Fl*/*Fl*^ system (VillinΔ*Mbd2*) (Supplementary Figure 9a) (35), where Mbd2 expression was reduced by >99%, suggesting a superior level of EC-Mbd2 deficiency (Supplementary Figure 9a) (6). Naïve VillinΔ*Mbd2* colon LP displayed equivalent myeloid cell proportions, total cell number and per cell IL-1β production, total IL-1β^+^ cell number and overall proportion of IL-1β^+^ myeloid populations to *Cre*^−^ control mice (Supplementary Figures 9b–f). As we observed in DSS treated *Mbd2*^−/−^ mice, CECs isolated from naïve VillinΔMbd2 mice showed significant dysregulation of genes associated with MHC-I and MHC-II (*Cd74, H2-Ab1, H2-Dmb1*), strongly suggesting that Mbd2 directly regulates CEC immunogenicity (Supplementary Figure 10).

**Figure 6:**
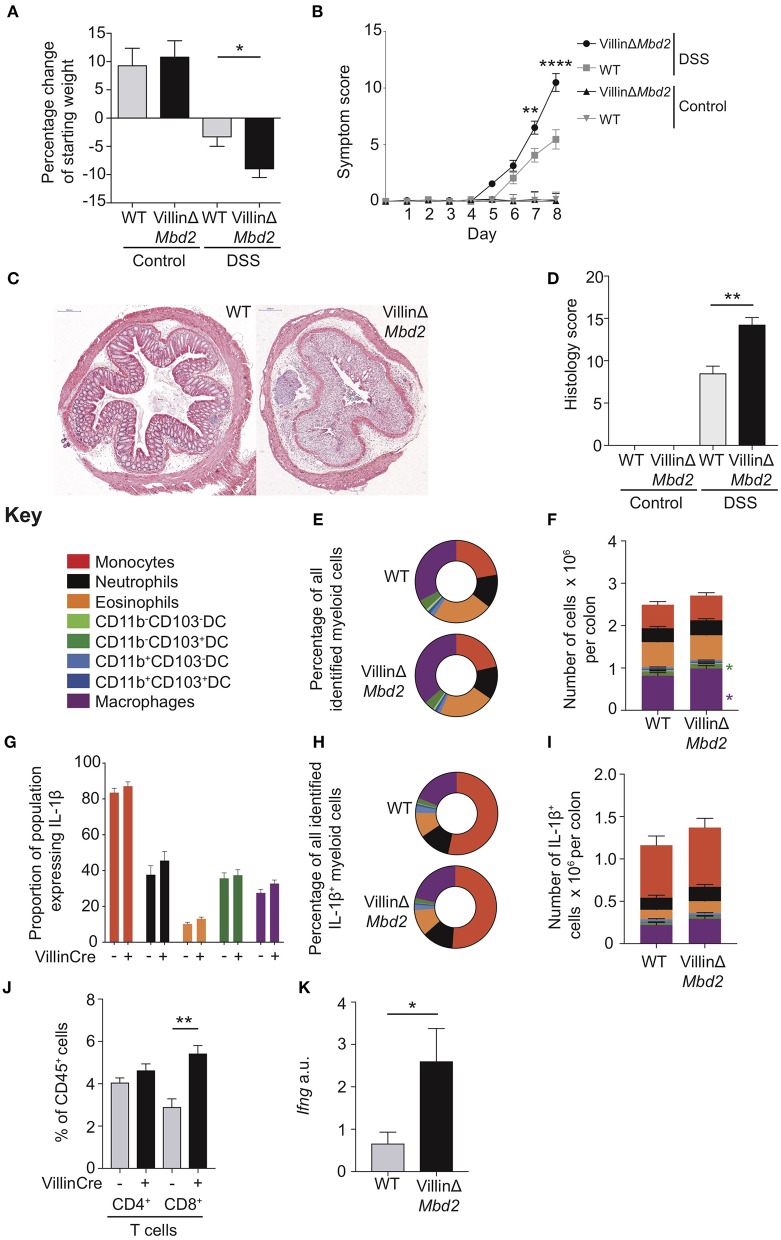
Epithelial cell expression of Mbd2 is vital to limit colitis. VillinΔ*Mbd2* or littermate *Cre*^−^ (WT) mice were co-housed and subjected to 2% DSS in drinking water, tissues were harvested 8 days post consecutive treatment. The severity of colitis in the mice was assessed by **(A)** weight loss following DSS treatment and **(B)** symptom score (calculated from daily assessment of weight loss, rectal bleeding and stool consistency). **(C)** colon sections obtained from DSS treated mice were stained with H&E, and **(D)** colitis severity was assessed blinded according to a DSS histo-pathology score. Colon lamina propria cells were isolated and the relative proportion and number of monocytes, neutrophils, eosinophils, macrophages, DC subsets **(E,F)** and T cells **(J)** were assessed by flow cytometry. Lamina propria cells were incubated for 3 h with 1 μl/ml Golgistop and **(G)** the proportion of monocyte, neutrophil, eosinophil, cDC1s (CD11b^−^CD103^+^), and macrophages that express IL-1β **(H)** the proportion of myeloid IL-1β^+^ cells, **(I)** and the total number of IL-1β^+^ cells was assessed by intracellular staining and flow cytometry. **(J)** Number of CD4^+^ and CD8α/β^+^ TCRα/β T cells as a proportion of CD45^+^ cells. **(K)** *ifng* mRNA levels derived from 1 cm sections of distal colon in DSS treated VillinCreΔ*Mbd2* or littermate *Cre*^−^ mice determined by qPCR relative to *gapdh*. All graphs show least mean square ±SEM, *n* = 15–25 per group, analyzed by linear regression of six independent experiments, except **(J,K)** which are representative data from three independent experiments. *P < 0.05, **P < 0.01, ****P < 0.0001.

Following DSS treatment, VillinΔ*Mbd2* mice developed more severe colitis than littermate *Cre*^−^ controls, as demonstrated by increased weight loss, symptom score and tissue architecture destruction (Figures 6A–D). However, in contrast to DSS-treated global *Mbd2*^−/−^ and CD11cΔ*Mbd2* mice, VillinΔ*Mbd2* animals had similar proportions of myeloid cells to WT controls and did not demonstrate either increased total numbers (Figures 6E,F), IL-1β^+^ populations (Figures 6G–I), or cDC1 CD103 expression (Supplementary Figure 11), that we observed in *Mbd2*^−/−^ and CD11cΔ*Mbd2* mice.

We have previously shown that DSS treated *Mbd2*^−/−^ colon LP CD4^+^ and CD8^+^ TCRα/β^+^ cells produce greater levels of IFN-γ, and therefore reasoned that increased *Mbd2*^−/−^ and VillinΔ*Mbd2* CEC MHC-II expression may affect T-cell function. Indeed, one of the most up-regulated genes in *Mbd2*^−/−^ CECs during DSS was the T-cell chemokine *Cxcl9* (Figure 5A), CEC sources of which have been shown to correlate with IBD severity (36). To support this, colon LP Mbd2 sufficient CD8α/β^+^ T cells from DSS treated VillinΔ*Mbd2* mice were significantly expanded, with corresponding increased levels of tissue IFN-γ transcript, mirroring our previously reported work in *Mbd2*^−/−^ mice (Figures 6J,K) (6). In summary, these data suggest that Mbd2 plays a dual role in limiting experimental colitis by regulating both CD11c-mediated myeloid cell, and CEC-mediated CD8^+^ T-cell, colon infiltration.

## Discussion

Intestinal surfaces are constantly exposed to a range of micro-organisms, requiring tight regulation of host cells to prevent over-exuberant immune responses that can lead to damaging inflammatory pathology (37). However, our understanding of how epigenetic processes affect immune-mediated intestinal inflammation and disease is currently limited (1). Here we show that expression of Mbd2, an epigenetic regulator of gene transcription, by both CD11c^+^ innate cells and ECs is crucial for limiting colonic inflammation.

In the steady state, *Mbd2*^−/−^ mice showed similar barrier integrity, absence of spontaneous inflammation and composition of the LP myeloid compartment to littermate controls (Figures 1A–C,E, Supplementary Figures 1c, 3). However, following epithelial disruption via DSS treatment, *Mbd2*^−/−^ mice displayed dramatically worse pathology, with severe weight loss, symptom score and immune infiltrate into the colon (Figures 1A–G). Indeed, the myeloid compartment of DSS treated *Mbd2*^−/−^ mice was defined by an enrichment of pro-inflammatory cell types, in particular an increased proportion of IL-1β expressing monocytes and neutrophils (Figures 1H–J). Monocytes are a key pro-inflammatory cell in human and murine colitis (18, 19, 38, 39), however colon monocytes isolated from *Mbd2*^−/−^ mice displayed dysregulation of only a handful of genes and similar per-cell cytokine producing capabilities compared to controls (Figures 2A,B,D). This suggested that increased tissue numbers of these cells represented a consequence, rather than cause, of increased colonic inflammation in *Mbd2*^−/−^ mice. We thus investigated other cellular sources of Mbd2 that could account for this exacerbated pathology.

cDC1s have been shown to express DC-specific transcription factors, migrate to MLNs in a CCR7 dependent manner, and interact with CECs to limit colitis pathlogy (10, 24), yet little is known about how epigenetic processes may regulate their function. We observed that *Mbd2*^−/−^ cDC1s significantly upregulated expression of *Ets2* (Figures 3A,B), a transcription factor that controls the induction of miR-155 (25, 40). miR-155 has been shown to be a potent pro-inflammatory mediator found at increased levels in the mucosa of IBD patients, suppressing SOCS1 and negative inhibitors of TLR4, thereby increasing IL-6 and IL-8 from immune cells including intestinal myofibroblasts (40, 41). Two other genes significantly upregulated in *Mbd2*^−/−^ cDC1s were *Gipc1* and *Adcy4* (Figures 3A,B), both of which can increase cellular cAMP^65−67^. Increases in cAMP can interfere with DC induction of Th2 responses, reduce MHC-II production and impair antigen presentation capabilities (27, 42). However, future dedicated investigation and validation of the function of these genes in DCs, and determining whether these are regulated by the action of Mbd2, may shed light on the downstream mechanisms that influence intestinal pathology during colitis.

Murine colon macrophages are important mediators of tolerance to the intestinal microbiota, with their depletion by removing Ccr2^+^ monocyte precursors resulting in increased susceptibility to chronic colitis (43). Comparison of WT and *Mbd2*^−/−^ cDC1 and macrophage gene expression revealed that both cell types displayed dysregulated expression of mediators associated with CEC crosstalk. Specifically, cDC1s from DSS treated *Mbd2*^−/−^ mice showed reduced levels of *Itgae*/CD103 [which interacts with CEC E-cadherin to prevent epithelial shedding and boost CEC maturation (28)] and *Nab1* [a co-repressor of early growth response 2 (Egr2) (44), defects in which result in reduced Stat5 signaling and increased susceptibility to γ-irradiation intestinal injury (29, 45)]. This was mirrored in reduced CD103 protein expression in *Mbd2*^−/−^ and CD11cΔ*Mbd2* cDC1s (Supplementary Figure 6). Furthermore, *Trem2* downregulation, which has been implicated in colonic epithelial repair during acute injury (32), was evident in *Mbd2*^−/−^ macrophages. Together, this suggested a role for Mbd2 in the regulation of gene expression in two important CD11c^+^ innate intestinal populations, with dysregulated expression of genes implicated in CEC crosstalk.

To test the relevance of these observations, we utilized CD11c-restricted Mbd2 depletion (CD11cΔ*Mbd2* mice), which resulted in increased susceptibility to DSS colitis compared to *Cre*^−^ controls (Figures 4A–D). Mechanistically, Mbd2 expression by CD11c^+^ cDC1s or macrophages regulated basic cellular processes such as antigen presentation and epithelial cross-talk, and CD11c-restricted Mbd2 depletion was accompanied by in an increase in IL-β^+^ monocyte recruitment. However, the severity of colitis observed in CD11cΔ*Mbd2* mice did not reach the same level as that evident in globally deficient *Mbd2*^−/−^ mice (Figures 1B,C), so we sought to assess the role for Mbd2 in CD11c^−^ cell types that might explain increased colitis susceptibility.

CECs regulate GI tract immunity by forming a physical barrier, producing anti-microbial products such as defensins and calprotectin (14), present antigen via MHC-II and secrete cytokines such as IL-10, TGF-β, and IL-12p70 (46). The differential expression of genes implicated in CEC-crosstalk from *Mbd2*^−/−^ cDC1s and macrophages (Figures 3A–D), suggested *Mbd2* may have direct and/or indirect roles in influencing CECs during colonic inflammation. Indeed, our mRNA expression dataset revealed that *Mbd2*^−/−^ CECs displayed marked up-regulation of genes associated with MHC-I and -II. MHC molecules are found at increased levels on CECs in active IBD, with MHC-II controlled by a transcriptional complex that includes the master transactivator, Ciita (47). Like Mbd2, the *Ciita* transcriptional regulator HIV Tat-interacting protein [HTAPIP, aka K(lysine) acetyltransferase 5 (Kat5)], alters histone acetylation, which in turn regulates chromatin remodeling, signal transduction, and Tat mediated MHC expression (48). In addition, EC deletion of histone deacteylase (Hdac) 1 and 2 or 3 in mice has been implicated in determining the overall pre-disposition to intestinal pathology (12, 35). We also observed reduced surface CD103 protein on cDC1s from *Mbd2*^−/−^ and CD11cΔ*Mbd2*, but not VillinΔ*Mbd2*, mice. CD103 (α_E_β_7_ integrin) affects lymphocyte dendrite formation and motility in a ligand dependent fashion, and interacts with E-cadherin on ECs, deletion in which results in spontaneous colonic inflammation and death due to increased EC shedding and lack of EC maturation (25). Thus, Mbd2 may be required to support local colon DC-EC interactions, through CD103-E-cadherin interactions in these respective cell types.

Lastly, we observed equivalence in pro-inflammatory myeloid cells in EC-restricted Mbd2 deficient colitis, but increased *Mbd2*^−/−^ CEC expression of T-cell chemokine *Cxcl9*. It is possible that the increased colitis severity evident in global *Mbd2*^−/−^ vs. CD11cΔ*Mbd2* or VillinΔ*Mbd2* mice was due to the additive pathology of IFN-γ^+^ T-cell recruitment driven by Mbd2-deficient ECs. In support of this, we observed expansion of CD8^+^ T-cells in DSS treated VillinΔ*Mbd2*, but not CD11cΔMbd2, where CECs were Mbd2 sufficient. Indeed, we have previously shown that Mbd2 deficient colon LP T cells may overexpress IFN-γ and IL-17 (6). Future co-culture experiments using Mbd2 sufficient/deficient CECs, T cells, DCs or macrophages would be a useful approach to address the relative importance of Mbd2 expression by each cell type in a more reductionist *in vitro* setting.

Thus, in a combined model of *Mbd2*^−/−^ colitis susceptibility (Graphical Abstract), we speculate that at the onset of intestinal inflammation, the Mbd2 deficient epithelium leads to further intestinal damage, through *Cxcl9* driven CD8^+^ T-cell recruitment and EC-MHC-II upregulation. Mbd2 deficient cDC1s facilitate this process by reduced CEC support (reduced *Itgae*/CD103 and Stat5 signaling) and expression of pro-inflammatory (mir-155 pathway) TFs. Mbd2 deficient macrophages compound reduced CEC repair (reduced *Trem2* expression), whilst simultaneously attempting to limit excessive inflammatory processes (increased *Ido1* and reduced *Retlna*). The end result is profound IL-1β^+^ monocyte accumulation facilitated by Mbd2 deficient CD11c^+^ cells, with simultaneous accumulation of CD8^+^ T-cells that increases tissue IFN-γ due to dysregulated, Mbd2 deficient, ECs.

In summary, Mbd2 is required for limiting colitis severity via the regulation of both myeloid and epithelial responses in the colon, employing multiple layers of cellular control that cumulatively prevent excessive inflammation in the intestines. Future work will focus on the role of DNA binding and chromatin accessibility in the ability of Mbd2 to regulate the function of cDC1s, macrophages and ECs. Strategies to target *Mbd2* for prevention of DC, macrophage/monocyte, or CEC exacerbation of intestinal inflammation may enable future development of innovative therapies to help control excessive inflammatory responses that define conditions such as IBD.

## Methods

### Mice

*Mbd2*^−/−^ and CD11cΔ*Mbd2* mice on a C57BL/6 background were bred in-house and maintained under specific pathogen-free conditions in the Faculty of Biology, Medicine and Health, University of Manchester, in compliance with the UK Home Office Animals (Scientific Procedures) Act 1986. VillinΔ*Mbd2* mice were generated by crossing *Mbd2*^*fl*/*fl*^ with *Villin-Cre*^+^ mice as previously described (2, 35). Age and sex matched mice (aged 8–22 weeks) were used in the experiments. CECs from VillinΔ*Mbd2* mice displayed a >98% reduction in *Mbd2* transcript compared to littermate *Cre*^−^ controls (Supplementary Figure 9a).

### DSS Model

Mice received 2% DSS salt b/w (reagent grade MW 36,000–50,000 kDa; MP Biomedicals) *ad libitum* in sterile drinking water for 6 days as described previously (49). Mice were monitored daily for change in weight, rectal bleeding and diarrhea to generate a symptom score. Experimental mice, wherever possible, were co-housed from birth with littermate controls. In histology analysis, the most distal 1 cm of colon was used for consistency between experiments, placed in 10% neutral buffered formalin for 24 h before being transferred to 20% ethanol. Samples were processed using standard hematoxylin and eosin (H&E) stain before being scored blinded using an established DSS histo-pathology protocol (50).

### Cell Isolation

LP cells were obtained from mouse colon by enzymatic digestion as described previously (49). Briefly the large intestines of mice were excised and soaked in ice cold PBS, before removing excess fat and feces, opened longitudinally and washed in Hank's balanced salt solution (HBSS; Sigma) 2% fetal calf serum (FCS; Sigma) before being cut into 0.5 cm sections. A further HBSS containing 2 mM EDTA (Gibco) wash step was used to remove mucous before digestion in complete RPMI (cRPMI) (2 mM L-glutamine{Gibco}, RPMI 1640, 100 μg/ml penicillin, 100 μg/ml streptomycin and 10% FCS, {all Sigma}) containing 0.5 U/ml Liberase TM and 0.1 mg/ml Type IV DNAse from bovine pancreas (both Sigma) for 45 min in a shaking incubator at 180 rpm, 37°C. Cells were then washed in cRPMI, passed through a 40-μM cell strainer (Thermo Fisher Scientific) with the aid of a syringe plunger before resuspension to the required concentration.

### Flow Cytometric Analysis and Sorting of Cells

Processed single cell suspensions [0.5–2 × 10^6^ cells per stain] were stained first using LiveDead blue (Invitrogen) at a 1:2,000 dilution in 10 μl PBS for 10 min at room temperature then FcR-Block (2.4G2), followed by the antibodies listed in Supplementary Table 3 at 4°C for 30 min, before being fixed in 1% paraformaldehyde prior to acquisition as described previously (51). To detect intracellular cytokines, cells were incubated in cRMPI at 37°C in 5% CO_2_ for 3 h in the presence of 1 μl/ml GolgiStop (BD Biosciences). For intracellular staining after fixation in 1% paraformaldehyde, cells were permeabilized using Cytofix/Cytoperm (BD Biosciences) or FoxP3 Transcription Factor buffer kit (ThermoFisher) and incubated with intracellular antibodies for 1 h at 4°C (Supplementary Table 3). Cells were washed prior to sample acquisition. All stained samples were acquired using a BD Fortessa (BD Biosciences) and analyzed using FlowJo v.9 software (TreeStar). Flow cytometer photomultiplier tube voltages were applied to ensure best compensation whilst aligning to the latest cytometer setup and tracking settings. The number of myeloid cells per mouse colon was determined by calculating the proportion of identified populations in Live/singlet/intact/CD45^+^/Lineage^−^ cells, myeloid population data was then expressed per mouse colon using the total LP cell count derived after purification.

Colon LP CD45^+^Ly6G^−^Siglec-F^−^CD11b^+^Ly6C^+^ monocytes, CD45^+^Ly6G^−^Siglec-F^−^CD11b^+^Ly6C^−^MHC-II^+^F4/80^+^ macrophages, CD45^+^Ly6G^−^Siglec-F^−^CD11b^−^CD11c^+^CD103^+^ cDC1s and EpCAM^+^CD45^−^CD3^−^F4/80^−^ from WT or *Mbd2*^−/−^ mice after 6 days of DSS treatment were FACS-purified using a BD Influx 70 μm nozzle size at 60PSI. Cell purity was confirmed at >95% before processing for RNA isolation and microarray analysis.

### mRNA Microarray and Quantitative PCR (qPCR)

For murine colon/spleen tissue samples or sorted cell populations total RNA was extracted using an adapted protocol (52) of the *mir*Vana miRNA isolation kit (Thermofisher). RNA quality was assessed using Agilent RNA 6000 Pico assay, RNA integrity number (RIN) >8.5 was considered satisfactory for subsequent microarray analysis. For microarrays, RNA was labeled using TotalPrep RNA amplification kits (Life Technologies) and hybridized with Illumina MouseWG-6BeadChip arrays with 2–5 biological replicates (1–10 mice per replicate) from three separate experiments of FACS-purified populations as described above. All analyses were conducted in R using Bioconductor. Pairwise group comparisons were undertaken using linear modeling. Subsequently, empirical Bayesian analysis was applied, including vertical (within a given comparison) *P*-value adjustment for multiple testing, which controls for false-discovery rate, using the limma Bioconductor package. An adjusted *P* < 0.01 and absolute log base2 fold change ≥1.0 was taken to screen candidate genes for further analysis. Functional enrichment analyses were performed for GO terms using the appropriate packages. Focused “genes of interest” lists were assembled from the literature and other publicly available resources.

To measure gene expression of whole tissue (1 cm section of distal colon or spleen was placed in 500 μl of RNALater and kept on dry ice before storage at −80°C) or purified cells by RT-qPCR, complementary DNA of extracted RNA was generated using SuperScript-III and Oligo-dT (Life Technologies). Relative quantification of genes of interest was performed by qPCR analysis using QuantStudio 12 Flex Real Time PCR system, with Fast SYBR® Green Master Mix (Life Technologies), compared with a serially diluted standard of pooled complementary DNA. Expression was normalized to *Gapdh*. Primers are listed in Supplementary Table 4.

### FITC Dextran Intestinal Permeability Assay

Naïve *Mbd2*^−/−^ and littermate controls were gavaged with 4 kDa FITC dextran at 60 mg/100 g body weight with serum obtained 4 h post-gavage, concentrations determined by spectrofluorophotometer (490/525 nm).

### Colon Explant Supernatant Analysis

One cm sections of distal colon were removed and incubated for 24 h in 1 ml cRPMI. Cytokine concentrations in culture supernatant was derived by cytokine bead array (BD biosciences).

### Statistical Analysis

Statistical analyses were carried out using GraphPad Prism v.7 or JMP v.12 (SAS Institute). The data were checked to confirm normality and that groups had equal variance. One-way analysis of variance (ANOVA) with Tukey's multiple comparison tests was employed to determine significant differences between sample groups. Results from these tests were reported as significant if *P* ≤ 0.05, with results from these tests shown as mean ±SEM. For some experiments statistical analysis was carried out using JMP, in which case data were analyzed using three-way full-factorial fit models to assess effects such as “genotype,” “treatment,” and “experiment” on the response variable of interest. This allowed the interaction between effects to be taken into account in addition to their impact on the response variable, which enabled experimental repeats to be pooled increasing the power of the analysis (53). The least squares mean results table from the three-way full-factorial analysis was used to test the contrast between specific experimental groups using a joint F-test. A difference between experimental groups was taken to be significant if the *P*-value (Prob > F) was ≤ 0.05, with results in graphs shown as least squares mean ± SEM.

## Data Availability Statement

The datasets generated for this study can be found in the GSE134282.

## Ethics Statement

All animal experiments were performed under review of the University of Edinburgh and/or Manchester bioresearch veterinary services and in compliance with the UK Home Office Animals (Scientific Procedures) Act 1986.

## Disclosure

The Manchester Collaborative Center for Inflammation Research is a joint venture between the University of Manchester and GSK.

## Author Contributions

G-RJ, PC, and AM designed the project and coordinated the experimental work. G-RJ, AP-A, SB, and PC carried out the experimental work. AI analyzed and helped the interpret the microarray data. AM conceived the project. AM and PC supervised the research. G-RJ, PC, and AM wrote the manuscript with valuable input from all other authors.

### Conflict of Interest

The authors declare that the research was conducted in the absence of any commercial or financial relationships that could be construed as a potential conflict of interest.
