# Supplementary figures and images for: The Methyl-CpG-Binding Protein Mbd2 Regulates Susceptibility to Experimental Colitis via Control of CD11c+ Cells and Colonic Epithelium
Source: Front Immunol. 2020 Feb 14;11:183. doi: 10.3389/fimmu.2020.00183 (PMC7033935; doi:10.3389/fimmu.2020.00183)

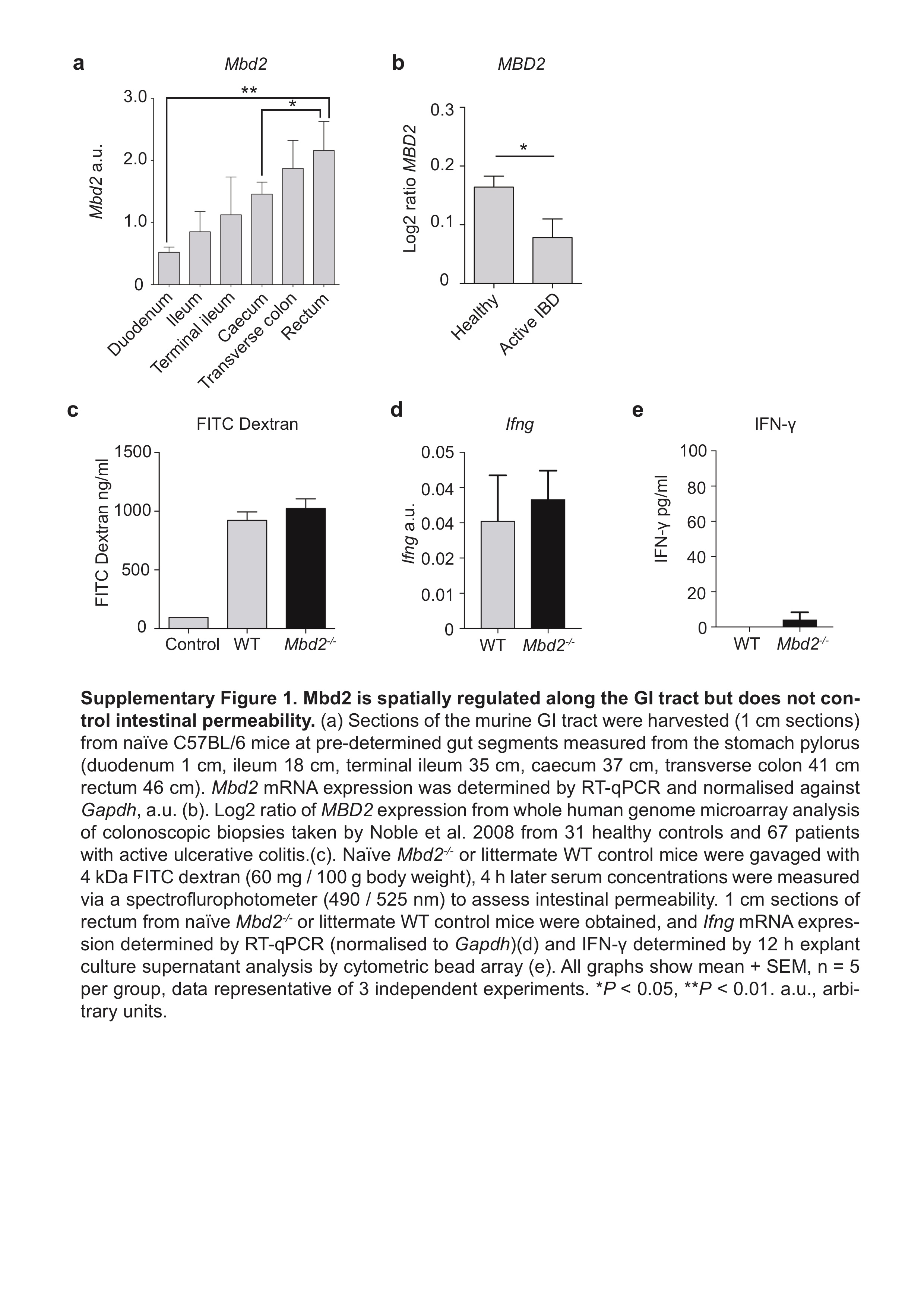

Supplement: Supplementary file 5 [file Image_1.jpg]

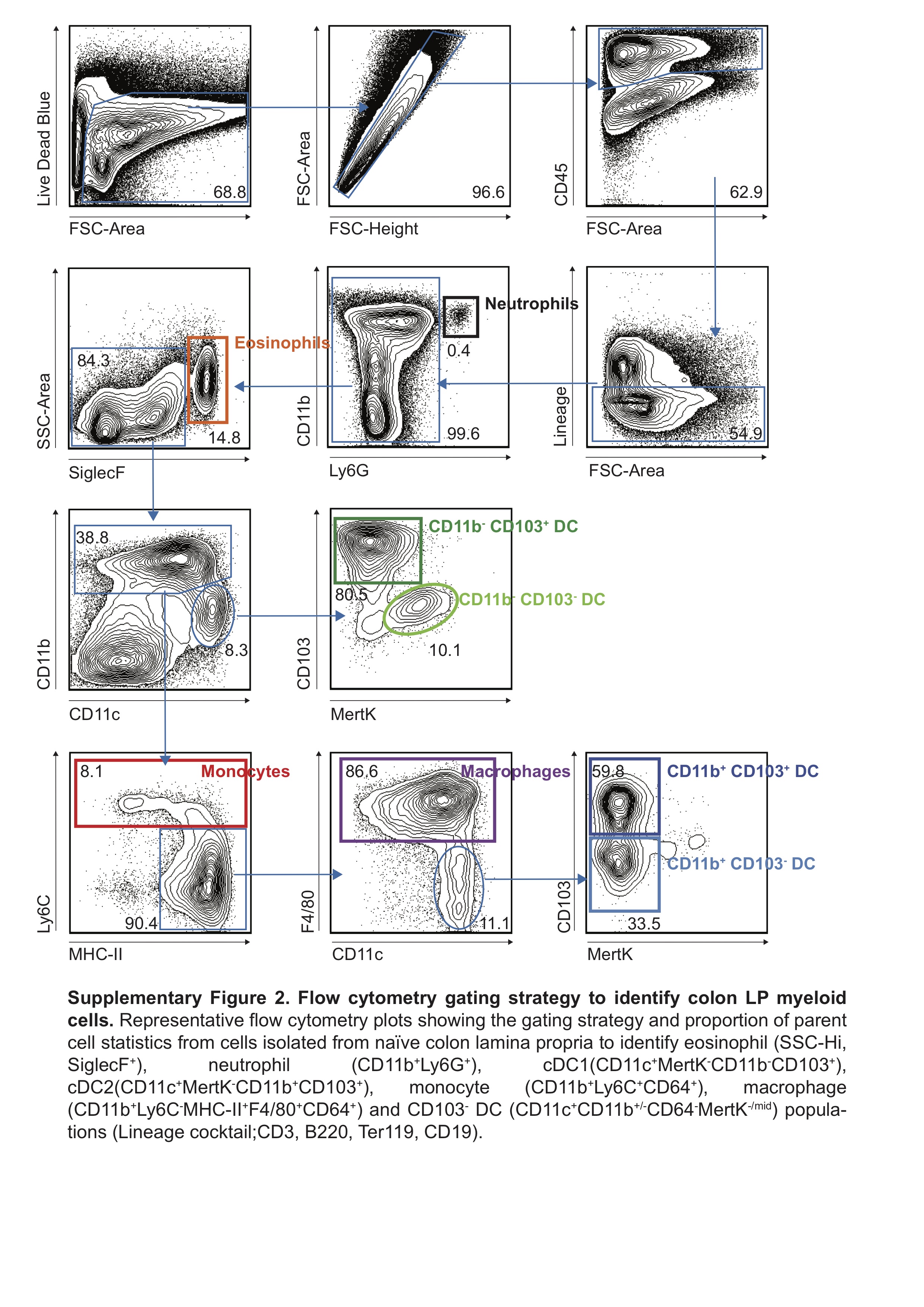

Supplement: Supplementary file 6 [file Image_2.JPEG]

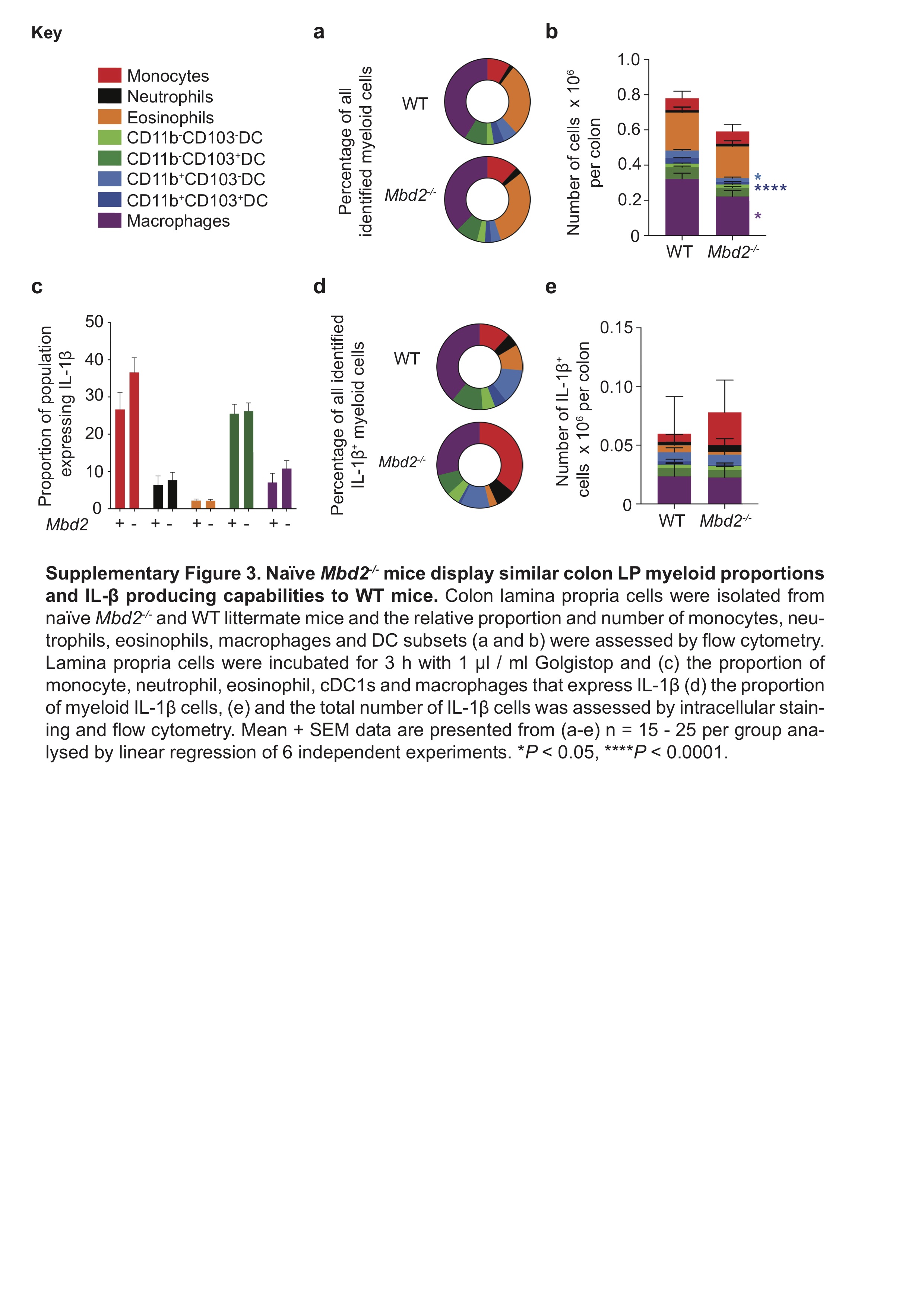

Supplement: Supplementary file 7 [file Image_3.jpg]

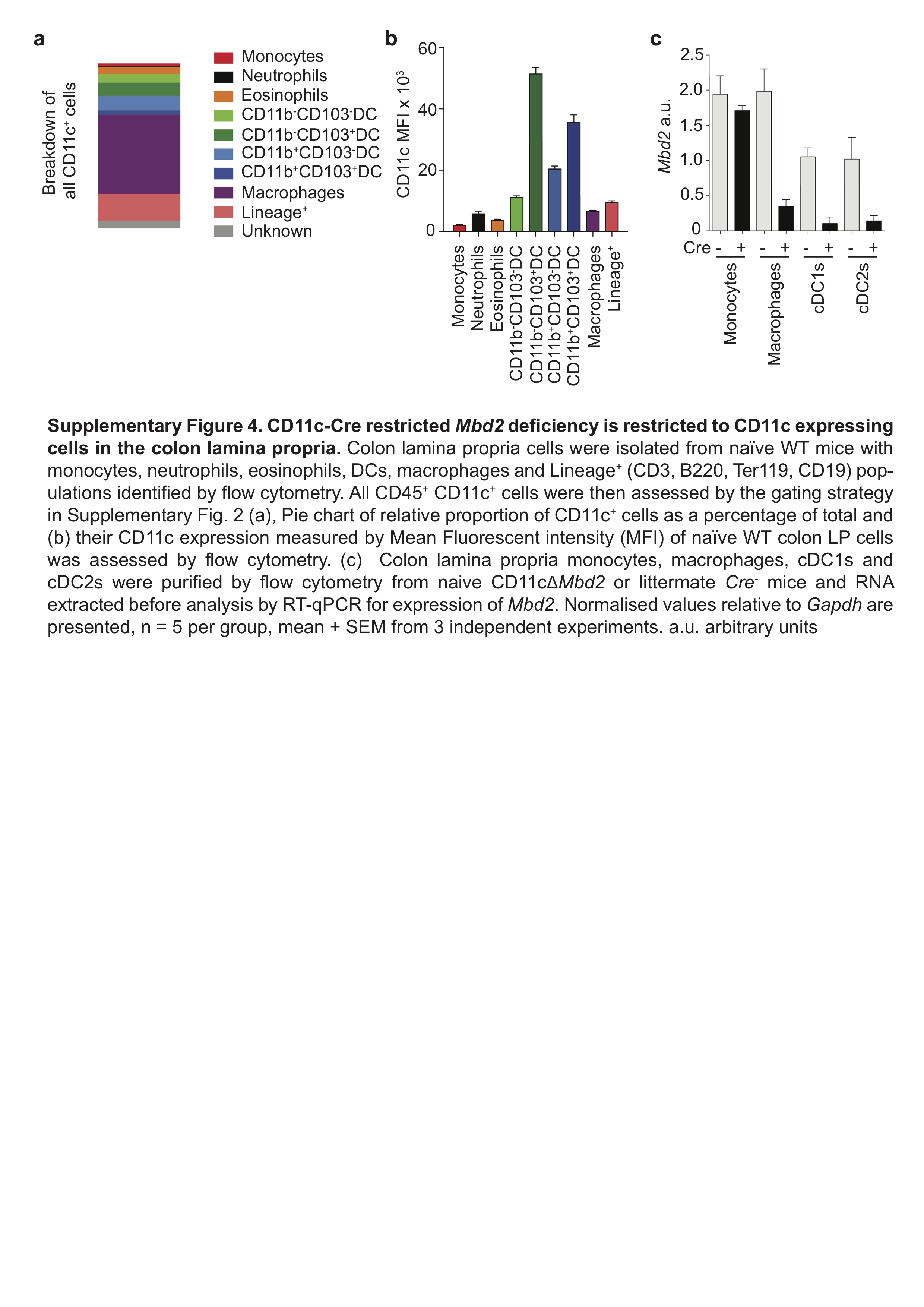

Supplement: Supplementary file 8 [file Image_4.jpg]

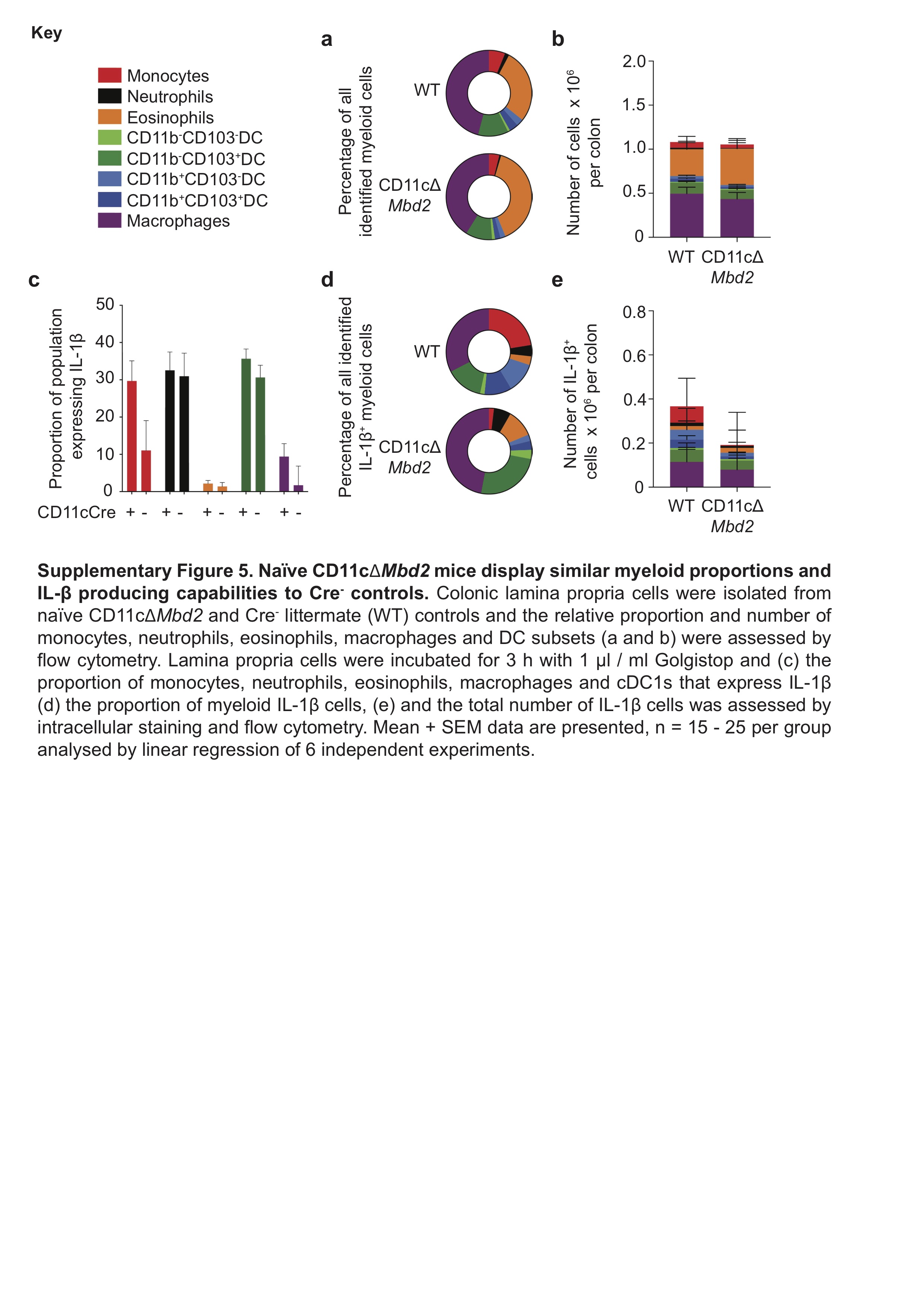

Supplement: Supplementary file 9 [file Image_5.jpg]

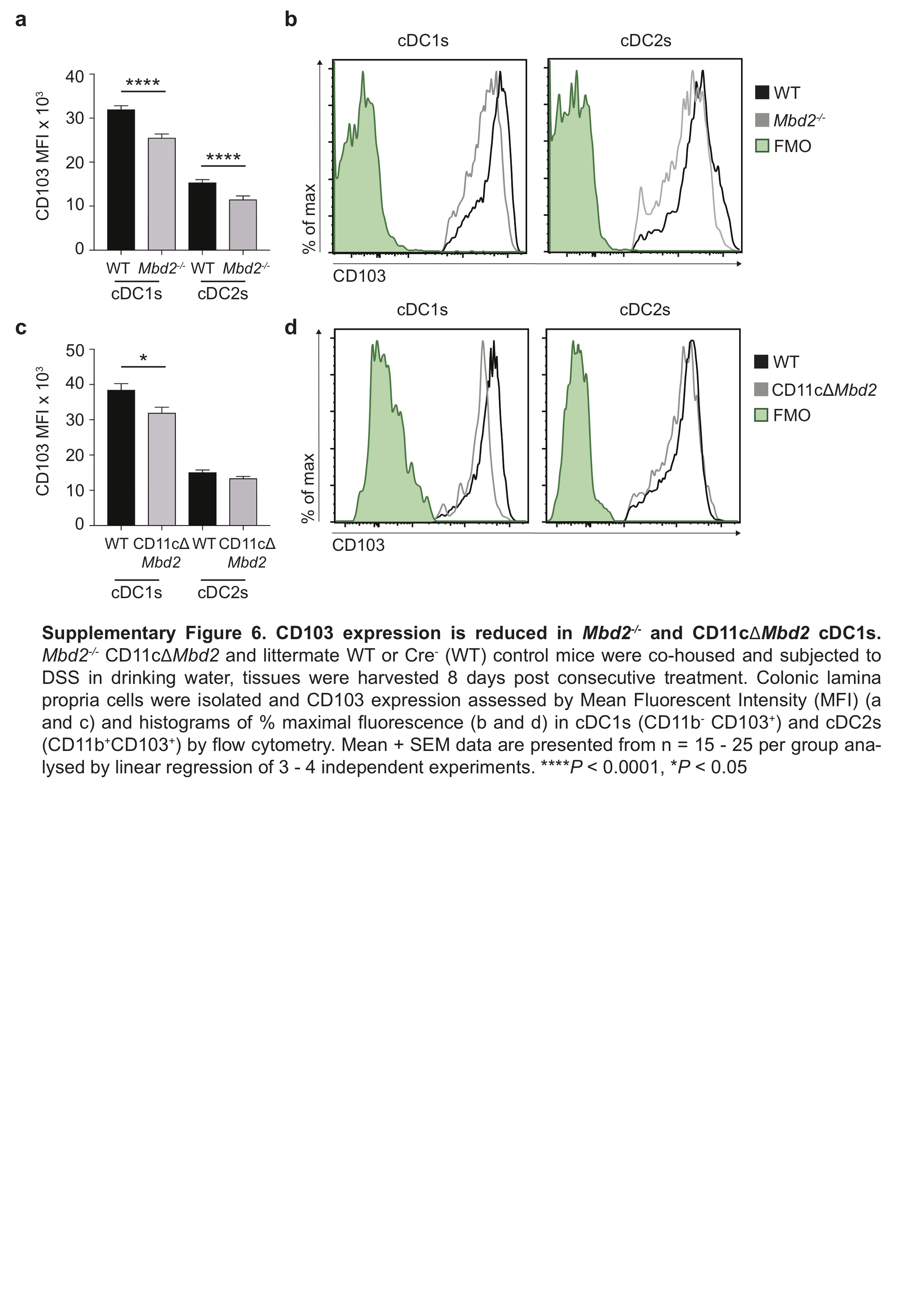

Supplement: Supplementary file 10 [file Image_6.jpg]

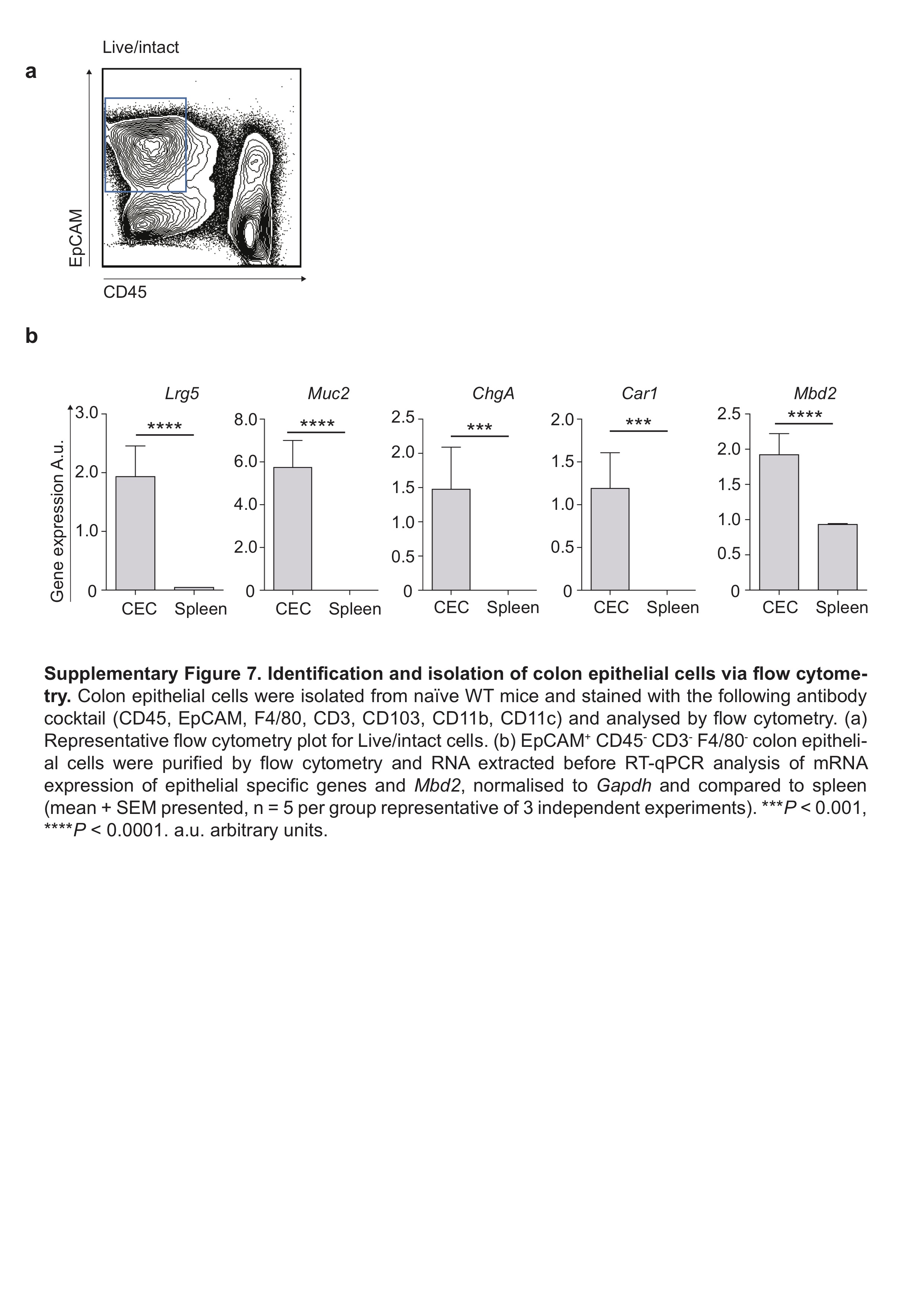

Supplement: Supplementary file 11 [file Image_7.jpg]

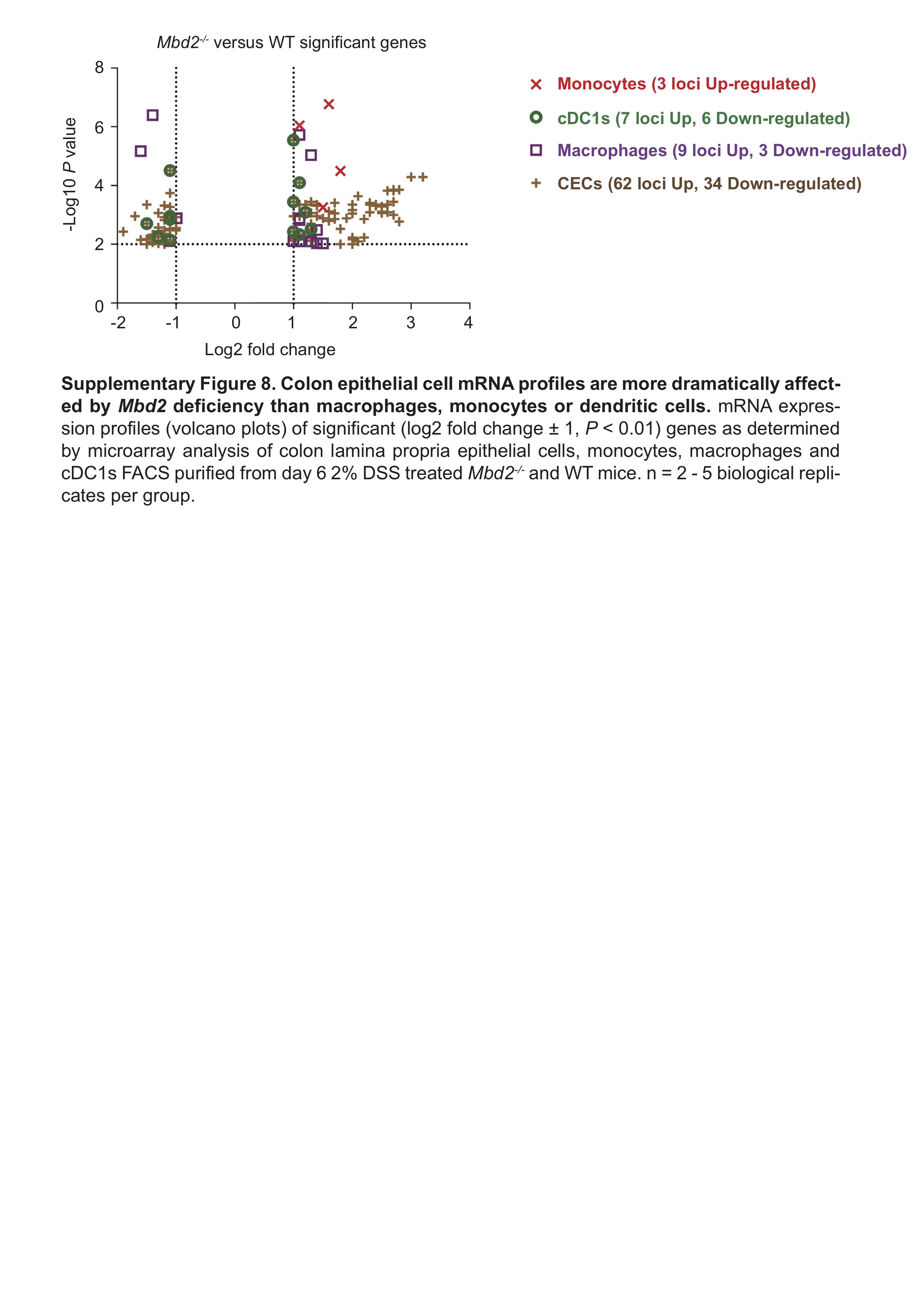

Supplement: Supplementary file 12 [file Image_8.jpg]

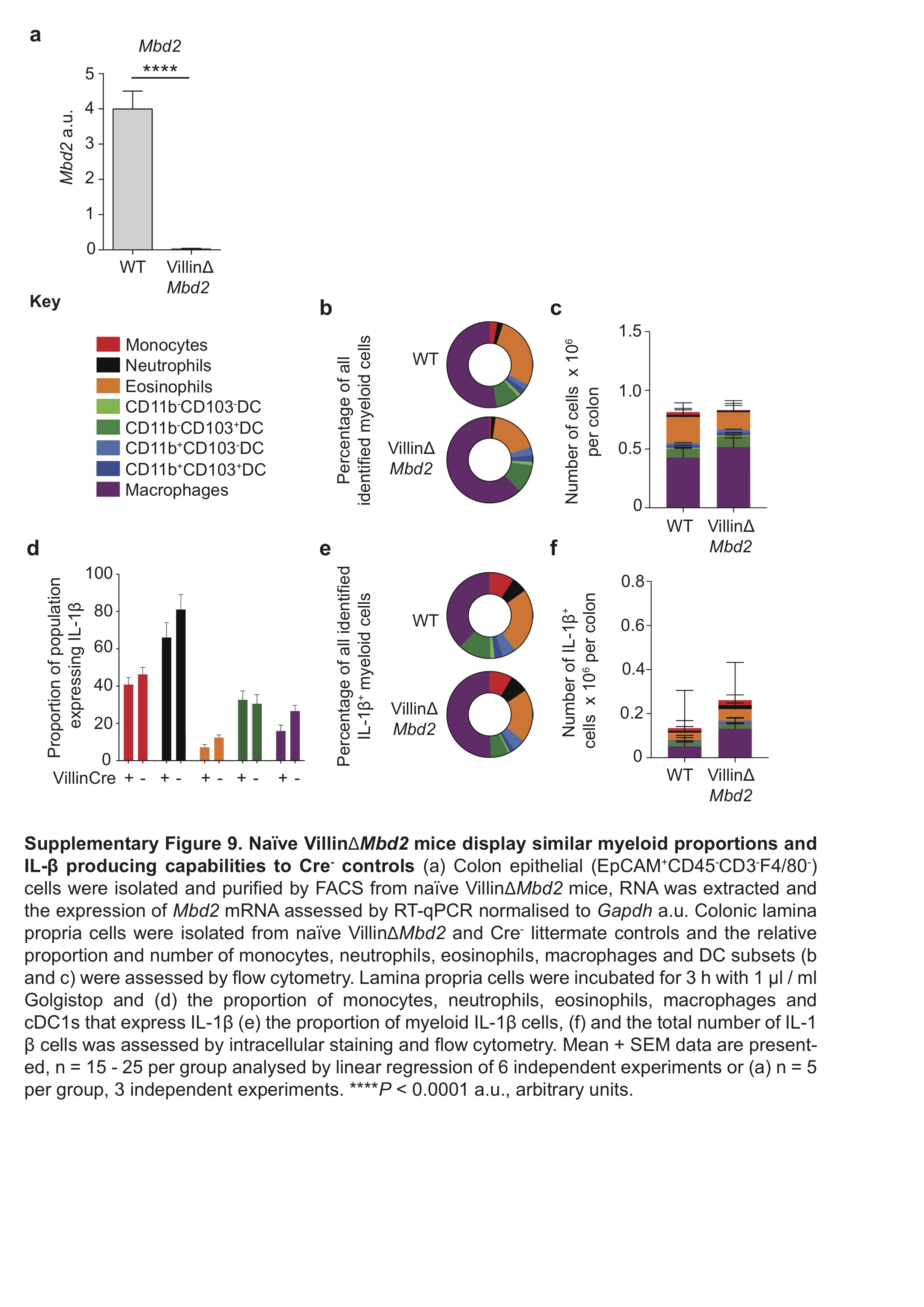

Supplement: Supplementary file 13 [file Image_9.jpg]

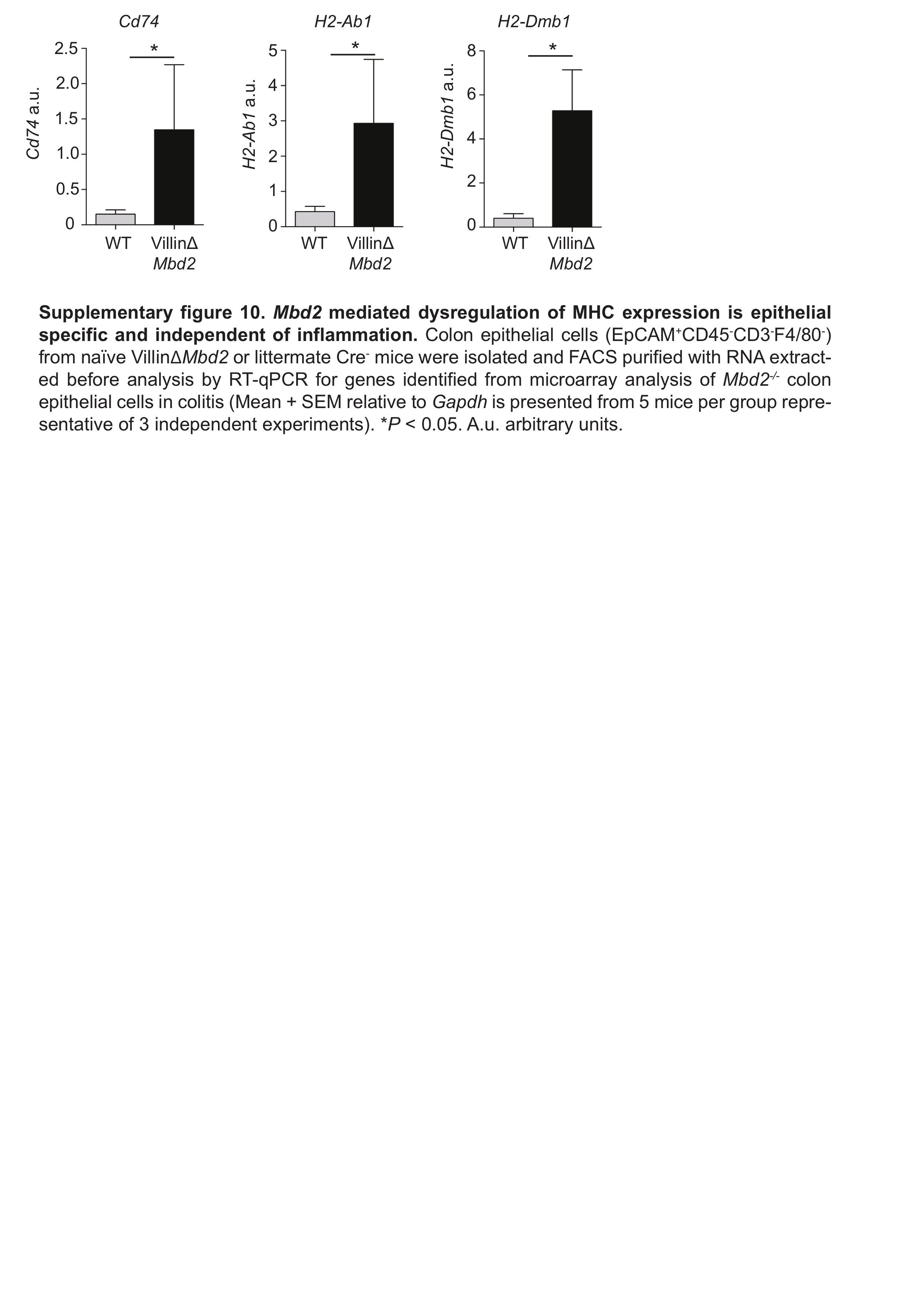

Supplement: Supplementary file 14 [file Image_10.jpg]

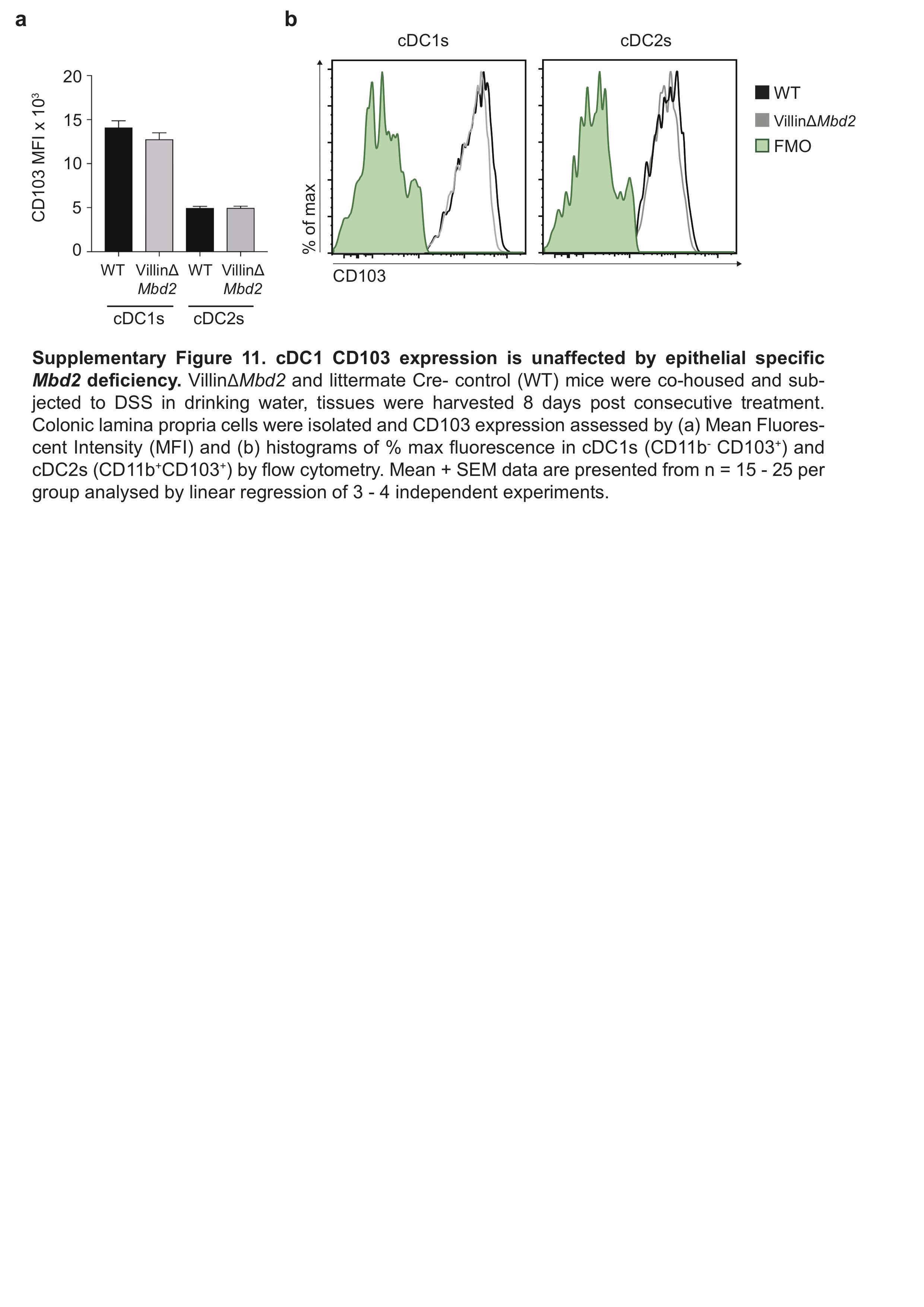

Supplement: Supplementary file 15 [file Image_11.jpg]
